# Robust Stability and Robust Stabilization of Discrete-Time Markov Jump Linear Systems Under a Class of Stochastic Structured Nonlinear Uncertainties

**DOI:** 10.3390/e27080858

**Published:** 2025-08-13

**Authors:** Vasile Dragan, Samir Aberkane

**Affiliations:** 1 Institute of Mathematics “Simion Stoilow” of the Romanian Academy, P.O. Box 1-764, 014700 Bucharest, Romania; vasile.dragan@imar.ro; 2 Academy of Romanian Scientists, 3 Ilfov, 050044 Bucharest, Romania; 3 Université de Lorraine, CNRS, CRAN, 54000 Nancy, France

**Keywords:** robust stability, Markov jump linear systems, stability radius, scaling

## Abstract

Robust stability/stabilization for discrete-time time-varying Markovian jump linear systems subject to block-diagonal stochastic parameter perturbations is addressed in this paper. Using a scaling technique, we succeed in effectively addressing the multi-perturbations case. We obtain an estimation of the lower bound of the stability radius in terms of the unique bounded and positive semidefinite solutions of adequately defined *parameterized* backward Lyapunov difference equations. In the time-invariant case, we show that such a lower bound is actually the exact value of the stability radius. Using the obtained result, we effectively address the state-feedback robust stabilization problem.

## 1. Introduction

The class of Markovian jump linear systems (MJLSs), a type of hybrid stochastic systems, is one of the most powerful modeling paradigm for systems facing random abrupt changes. Problems such as moment stability, sample path stability, and optimal and robust control, along with important applications of such systems, can be found in several references in the current literature, for instance [1,2,3,4] and the references therein.

In this paper, we study the robust stability and robust stabilization problems under multi-perturbations for a class of discrete-time time-varying MJLSs affected by parametric uncertainties of multiplicative white noise type with unknown intensity. The problem of robustness with respect to stochastic parametric uncertainties is a central one in control theory. This is mainly motivated by considerations around engineering applications. One of the most powerful tools that has been developed to tackle robustness issues is the concept of the stability radii, which, as coined by [5], lies at the heart of robustness analysis problems and as such constitute an area of central interest. Hence, since the seminal work of D. Hinrichsen and A.J. Pritchard ([6,7]), this has been a very active research domain.

In the MJLS context, one can cite the seminal reference of [8]. For recent developments on the topic, we refer to [9,10]. For Itô-type stochastic systems, we cite [11,12]. Very recent references such as [13] illustrate that this domain remains active. In order to cope with the multi-perturbations case, we use a scaling technique (see [14,15]). This allows us to obtain an estimation of the lower bound of the stability radius. The main contributions of this article can be summarized as follows:

- We first provide a lower bound of the stability radius in terms of the unique bounded and positive semidefinite solutions of adequately defined *parameterized* backward Lyapunov difference equations (Theorem 2).
- In the time-invariant case, we proceed to show that such a lower bound is actually the exact value of the stability radius (Theorem 3).
- The problem of state-feedback robust stabilization is also addressed. A first solution is obtained in Theorem 4, which is stated in terms of the solution of an adequately defined parameterized nonlinear matrix inequality.
- Using the results in Theorem 4, we proceed to obtain a numerically tractable condition for the computation of a state-feedback robust stabilizing controller. The feedback gain is constructed on the basis of the stabilizing solution of an adequately defined discrete-time algebraic Riccati equation.

The rest of this article is organized as follows: Section 2 provides some preliminary definitions and introduces the problem formulation. The main results are established in Section 3 for the time-varying case and in Section 4 for the time-invariant case. The robust stabilization problem is addressed in Section 5. Finally, a numerical example is provided in Section 6 to illustrate the theoretical results.

**Notation**: $A^{⊤}$ stands for the transpose of the matrix *A*. In block matrices, ★ indicates symmetric terms: $\left(\begin{array}{cc} A & B\\ B^{T} & C \end{array}\right)=\left(\begin{array}{cc} A & ★\\ B^{T} & C \end{array}\right)=\left(\begin{array}{cc} A & B\\ ★ & C \end{array}\right)$. The expression MN★ is equivalent to $MNM^{T}$, while M★ is equivalent to $MM^{T}$.

## 2. Problem Formulation and Preliminaries

### 2.1. Model Description

Consider the system $G_{\Delta }$ with the state space representation described by

(1a) $$\begin{array}{cc} \; & x\left(t+1\right)=A\left(t,\theta_{t}\right)x\left(t\right)+\sum_{k=1}^{r}w_{k}\left(t\right)D_{k}\left(t,\theta_{t}\right)\Delta_{k}\left(t,z_{k}\left(t\right),\theta_{t}\right), \end{array}$$

(1b) $$\begin{array}{cc} \; & z_{k}\left(t\right)=C_{k}\left(t,\theta_{t}\right)x\left(t\right),\;1\leq k\leq r, \end{array}$$

where $x\left(t\right)\in R^{n}$ is the system state vector, ${\left\{w_{t}\right\}}_{t\geq 0}$, $\left(w_{t}={\left(w_{1}\left(t\right),\cdots ,w_{r}\left(t\right)\right)}^{T}\right)$ is a sequence of independent random vectors, and the triple $\left({\left\{\theta_{t}\right\}}_{t\geq 0},{\left\{P_{t}\right\}}_{t\geq 0},\Theta \right)$ is a time non-homogeneous Markov chain defined on a given probability space (Ω,F,P) with the finite states set Θ={1,2,…,N} and the sequence of transition probability matrices ${\left\{P_{t}\right\}}_{t\geq 0}$. In (1), $A\left(t,i\right)\in R^{n\times n},D_{k}\left(t,i\right)\in R^{n\times m_{k}},C_{k}\left(t,i\right)\in R^{\nu_{k}\times n},1\leq k\leq r,\left(t,i\right)\in Z_{+}\times \Theta$ are known given matrices, while $z_{k}\to \Delta_{k}\left(t,z_{k},i\right):R^{\nu_{k}}\to R^{m_{k}}$ are arbitrary measurable functions with the additional properties

(2) $$\begin{array}{c} \Delta_{k}\left(t,0,i\right)=0,\;\forall \;\;\left(t,i\right)\in Z_{+}\times \Theta \end{array}$$

and

(3) $$\begin{array}{c} |\Delta_{k}\left(t,z_{k},i\right)|\leq \gamma_{k}\left|z_{k}\right|,\;\forall \;\left(t,z_{k},i\right)\in Z_{+}\times R^{\nu_{k}}\times \Theta ,\;k\in \underline{r} \end{array}$$

with $\underline{r}:=\left\{1,2,\ldots ,r\right\}$. In (3), $\gamma_{k}>0$ does not depend upon $\left(t,z_{k},i\right)$ but can depend upon the function $\Delta_{k}\left(t,\cdot ,i\right)$.

Throughout this paper, |·| stands for the Euclidean norm of a vector, i.e., ${\left|x\right|}^{2}=x^{⊤}x$.

**Remark** **1.** *Under a condition of type (2), the linear growth condition of type (3) seems to be more relaxed than a condition of Lipschitz type, such as the one considered in [16] in the continuous time case. However, the measurability property of the functions $\Delta_{k}\left(t,\cdot ,i\right)$ is required in order to be sure that the solutions of the perturbed system (1) are stochastic processes adapted to the filtration generated by the stochastic processes ${\left\{w\left(t\right)\right\}}_{t\geq 0}$ and ${\left\{\theta_{t}\right\}}_{t\geq 0}$ involved in (1).*

We denote $\mathrm{NL}(R^{\nu_{k}},R^{m_{k}})$ as the set of all measurable functions $z_{k}\to \Delta_{k}\left(t,z_{k},i\right):R^{\nu_{k}}\to R^{m_{k}}$ which satisfy conditions of the form (2)–(3). To ease the presentation, we shall denote as $\Delta_{k}\left(\cdot \right)$ the elements of the set $\mathrm{NL}(R^{\nu_{k}},R^{m_{k}})$. This allows us to describe the set of admissible uncertainties as

(4) $$\begin{array}{c} D=\{△\left(\cdot \right)=\left(\Delta_{1}\left(\cdot \right),\Delta_{2}\left(\cdot \right),\ldots ,\Delta_{r}\left(\cdot \right)\right)\;|\;\Delta_{k}\left(\cdot \right)\in \mathrm{NL}\left(R^{\nu_{k}},R^{m_{k}}\right),k\in \underline{r}\}. \end{array}$$

The system (1) may be regarded as a perturbation of the nominal system

(5) $$\begin{array}{c} x\left(t+1\right)=A\left(t,\theta_{t}\right)x\left(t\right). \end{array}$$

The term $\sum_{k=1}^{r}w_{k}\left(t\right)D_{k}\left(t,\theta_{t}\right)\Delta_{k}\left(t,z_{k}\left(t\right),\theta_{t}\right)$ models the possible parametric excitations of white noise type of the coefficients of the nominal system (5), for which the magnitude is in general unknown.

Consider the space of admissible perturbations D defined in (4) and let us assume that the zero solution of the nominal system (5) is exponentially stable in mean square (in a sense which will be defined bellow). The first problem we address in this paper is the characterization of a *wide* set D⊂D of perturbations △(·) with the property that the perturbed system $G_{\Delta }$ is exponentially stable in the mean square for all △(·)∈D. For a precise description of the set D, we shall use the notion of the stability radius. To this end, we shall introduce a norm on the space of admissible perturbations D.

### 2.2. Basic Assumptions

In order to provide precise definitions of the concept of exponential stability in the mean square invoked in this paper, let us consider the following discrete-time linear stochastic system:

(6) $$G:\;x\left(t+1\right)=\left(A_{0}\left(t,\theta_{t}\right)+\overset{r}{\sum_{k=1}}w_{k}\left(t\right)A_{k}\left(t,\theta_{t}\right)\right)x\left(t\right)$$

where $x\left(t\right)\in R^{n}$ is the system state vector, while ${\left\{w_{t}\right\}}_{t\geq 0}$ and $\left({\left\{\theta_{t}\right\}}_{t\geq 0},{\left\{P_{t}\right\}}_{t\geq 0},\Theta \right)$ are defined as in the previous section.

Concerning the processes ${\left\{\theta_{t}\right\}}_{t\geq 0}$ and ${\left\{w\left(t\right)\right\}}_{t\geq 0}$, the following assumptions are made:

**(H1)** 
${\left\{w\left(t\right)\right\}}_{t\geq 0}$ is a sequence of independent random vectors with the following properties:
  $$E\left[w(t)\right]=0,\;E\left[w\left(t\right)w^{⊤}\left(t\right)\right]=I_{r},\;t\geq 0,$$
  $I_{r}$ being the identity matrix of size *r*. As usual, throughout this paper $E\left[\cdot \right]$ stands for the mathematical expectation and the superscript ⊤ denotes the transposition of a vector or a matrix.
**(H2)** 
For each t≥0, the σ-algebra $F_{t}$ is independent of the σ-algebra $G_{t}$, where $F_{t}=\sigma \left(w\left(s\right);0\leq s\leq t\right)$ and $G_{t}=\sigma \left(\theta_{s};0\leq s\leq t\right)$.
**(H3)** 
  (i) The transition probability matrices $P_{t},\;t\geq 0$ are nondegenerate stochastic matrices, meaning that their elements $p_{t}\left(i,j\right)$ have the properties $0\leq p_{t}\left(i,j\right)\leq 1$, $\sum_{\ell =1}^{N}p_{t}\left(i,\ell \right)=1$ and $\sum_{\ell =1}^{N}p_{t}\left(\ell ,j\right)>0$ for all i,j∈Θ.
  (ii) $\pi_{0}\left(i\right)=P\left\{\theta_{0}=i\right\}>0,\;1\leq i\leq N$.
  Note that in the developments of this note, the Markov chain is not prefixed, but it is assumed that the initial probability distributions $\pi_{0}=\left(\pi_{0}\left(1\right),\ldots ,\pi_{0}\left(N\right)\right)$ lie in the subset
  $$M_{N}=\left\{\pi_{0}|\pi_{0}\left(i\right)>0,1\leq i\leq N,\sum_{i=1}^{N}\pi_{0}\left(i\right)=1\right\}.$$
  It is worth mentioning that it is possible to check inductively that, under assumption (**H3**),
  $P\{\theta_{t}=i\}>0$ for all t>0,i∈Θ.
**(H4)** 
There exists an initial probability distribution ${\tilde{\pi }}_{0}\in M_{N}$ such that, for the Markov chain $\left({\left\{{\tilde{\theta }}_{t}\right\}}_{t\geq 0},{\left\{P_{t}\right\}}_{t\geq 0},\Theta \right)$ (with initial probability distribution ${\tilde{\pi }}_{0}$), there exists δ>0 such that
  (7) $${\tilde{\pi }}_{t}\left(i\right)=P\left\{{\tilde{\theta }}_{t}=i\right\}\geq \delta ,\;t\geq 0,\;i\in \Theta .$$

**(H5)** 
${\left\{A\left(t,i\right)\right\}}_{t\geq 0},{\left\{D_{k}\left(t,i\right)\right\}}_{t\geq 0},{\left\{C_{k}\left(t,i\right)\right\}}_{t\geq 0},1\leq k\leq r$ are fixed matrix-valued bounded sequences.

### 2.3. Lyapunov-Type Operators

Let $S_{n}\subset R^{n\times n}$ be the linear subspace of real symmetric matrices. Set $S_{n}^{N}=\underset{N\;\mathrm{times}}{\underset{︸}{S_{n}\times S_{n}\times \cdots \times S_{n}}}$.

$S_{n}^{N}$ is a real-ordered Hilbert space. The usual inner product on $S_{n}^{N}$ is

(8) $$\langle X,Y\rangle =\sum_{i=1}^{N}\mathrm{Tr}\left(X\left(i\right)Y\left(i\right)\right)$$

for all X=(X(1),…,X(N)) and Y=(Y(1),…,Y(N)) in $S_{n}^{N}$. On the space $S_{n}^{N}$, we consider the order relation ⪰ induced by the convex cone $S_{n}^{N+}=\left\{X\in S_{n}^{N}\;|\;X=\left(X\left(1\right),X\left(2\right),\ldots ,X\left(N\right)\right),X\left(i\right)\geq 0,1\leq i\leq N\right\}$. If $Y\left(\cdot \right):Z_{+}\to S_{n}^{N}$, we shall write $Y\left(t\right)≻≻0,t\in Z_{+}$ if there exists ε∈(0,∞) such that $Y\left(t,i\right)\geq \varepsilon I_{n}$ for all $\left(t,i\right)\in Z_{+}\times \Theta$. We shall write $Y\left(t\right)≺≺0,t\in Z_{+}$ iff $-Y\left(t\right)≻≻0,t\in Z_{+}$.

Based on the sequences ${\left\{A\left(t,i\right)\right\}}_{t\geq 0}$, i∈Θ, and ${\left\{P_{t}\right\}}_{t\geq 0}$ introduced previously, we define the following linear operators:

(9) $$\begin{array}{c} L_{t}:\;S_{n}^{N}\;⟶S_{n}^{N},\;S\;⟼L_{t}S=\left(\left(L_{t}S\right)\left(1\right),\ldots ,\left(L_{t}S\right)\left(N\right)\right) \end{array}$$

where

(10) $$\left(L_{t}S\right)\left(i\right)=\sum_{j=1}^{N}p_{t}\left(j,i\right)A\left(t,j\right)S\left(j\right)A^{T}\left(t,j\right).$$

The operators $L_{t}$ are called the Lyapunov-type operators associated with the nominal system (5). The adjoint operator of $L_{t}$ with respect to the inner product (8) is denoted $L_{t}^{*}$ and described by

(11) $$\begin{array}{c} \left(L_{t}^{*}S\right)\left(i\right)=\sum_{j=1}^{N}p_{t}\left(i,j\right)A^{T}\left(t,i\right)S\left(j\right)A\left(t,i\right),\;i\in \Theta . \end{array}$$

Let R(t,s) be the linear evolution operator defined on $S_{n}^{N}$ by the sequence ${\left\{L_{t}\right\}}_{t\geq 1}$, as follows:

(12) $$R\left(t,s\right)=\left\{\begin{array}{c} L_{t-1}L_{t-2}\cdots L_{s}\;\mathrm{if}\;t>s\geq 0\\ I_{S_{n}^{N}}\;\;\;\;\;\;\mathrm{if}\;t=s \end{array}\right.$$

where $I_{S_{n}^{N}}$ is the identity operator on $S_{n}^{N}$. Regarding systems of type (5), this refers to the following kinds of exponential stability in the mean square of the zero equilibrium.

**Definition** **1.** *We say that the zero solution of the nominal system (5) is:*

*(i) **Strong exponentially-stable in mean square** (SESMS) if the forward discrete-time linear equation on $S_{n}^{N}$: $X\left(t+1\right)=L_{t}X\left(t\right)$ is exponentially stable;*

*(ii) **Exponentially stable in mean square with conditioning** (ESMS-C) if there exist β≥1, q∈(0,1) such that*

(13) $$\begin{array}{c} E[|x\left(t;t_{0},x_{0}\right){|}^{2}\;|\;\theta_{t_{0}}=i]\leq \beta q^{t-t_{0}}{\left|x_{0}\right|}^{2} \end{array}$$

*for all $t\geq t_{0}\in Z_{+},x_{0}\in R^{n},i\in \Theta$ and any initial probability distribution $\pi_{0}$ of the Markov chain;*

*(iii) **Exponentially stable in mean square** (ESMS) if there exist β≥1, q∈(0,1) such that*

(14) $$\begin{array}{c} E[|x\left(t;t_{0},x_{0}\right){|}^{2}]\leq \beta q^{t-t_{0}}{\left|x_{0}\right|}^{2} \end{array}$$

*for all $t\geq t_{0}\in Z_{+},x_{0}\in R^{n}$ and any initial probability distribution $\pi_{0}$ of the Markov chain.*

In (13) and (14), $t\to x(t;t_{0},x_{0})$ is the solution of the nominal system which satisfies $x\left(t_{0};t_{0},x_{0}\right)=x_{0}$.

**Remark** **2.** *In general, in the time-varying context, the three kinds of exponential stability of the nominal system (5) introduced in Definition 1 are not equivalent. Only the implications (i)→(ii)→(iii) hold. If assumption (**H3**) is fulfilled, then (i)↔(ii), and if assumptions (**H3**) and (**H4**) are both satisfied, then the three kinds of exponential stability in the mean square introduced in the above definition are equivalent. More details may be found in Chapter 3 of [3].*

Regarding the exponential stability in the mean square of the equilibrium x=0 of the perturbed system in (1), we recall the following definition.

**Definition** **2.** *We say that the state x=0 of the perturbed system (1) is:*

*(i) **Globally exponentially stable in mean square with conditioning** (GESMS-C) if its solutions $x(t;t_{0},x_{0})$ have an upper bound as that from (13) for all $t\geq t_{0}\in Z_{+},i\in \Theta ,x_{0}\in R^{n}$ and any initial probability distribution of the Markov chain;*

*(ii) **Globally mean square stable with conditioning** (GMSS-C) if its solutions $x(t;t_{0},x_{0})$ satisfy*

(15) $$\begin{array}{c} \sum_{t=t_{0}}^{\infty }E[|x\left(t;t_{0},x_{0}\right){|}^{2}\;|\;\theta_{t_{0}}=i]\leq c|x_{0}{|}^{2} \end{array}$$

*for all $t_{0}\geq 0,x_{0}\in R^{n},i\in \Theta$ and any initial probability distribution $\pi_{0}$ of the Markov chain, c>0 being a constant not depending upon $t_{0},x_{0},i$, and $\pi_{0}$.*

**Remark** **3.** *It is obvious that the system in (1) is GMSS-C if it is GESMS-C. If assumptions (**H1**)–(**H3**) and (**H5**) are fulfilled, then the two kinds of stability of the zero solution of the system (1) are equivalent (see, e.g., Theorem 3.2 from [17]).*

## 3. Stability Radius

Before providing the definition of the stability radius, we first introduce a norm in the set of the uncertainties. If $\Delta_{k}\left(\cdot \right)={\left\{\Delta_{k}\left(t,\cdot ,i\right)\right\}}_{\left(t,i\right)\in Z_{+}\times \Theta }\in \mathrm{NL}\left(R^{\nu_{k}},R^{m_{k}}\right)$, we set:

(16) $$\begin{array}{c} |\Delta_{k}\left(\cdot \right)|=inf\{\gamma_{k}>0\;|\;|\Delta_{k}\left(t,z_{k},i\right)|\leq \gamma_{k}|z_{k}\left|,\forall \left(t,z_{k},i\right)\in Z_{+}\times R^{\nu_{k}}\times \Theta \right\}. \end{array}$$

Now, we may define the norm

(17) $$\begin{array}{c} ∥△\left(\cdot \right)∥=max_{k\in \underline{r}}\left|\Delta_{k}\left(\cdot \right)\right| \end{array}$$

for all $△\left(\cdot \right)=\left(\Delta_{1}\left(\cdot \right),\Delta_{2}\left(\cdot \right),\cdots ,\Delta_{r}\left(\cdot \right)\right)\in D$.

**Remark** **4.** *Among the admissible uncertainties from D we mention the linear uncertainties. These are of the form $△\left(\cdot \right)=\left(\Delta_{1}\left(\cdot \right),\Delta_{2}\left(\cdot \right),\cdots ,\Delta_{r}\left(\cdot \right)\right)$ with $\Delta_{k}\left(t,z_{k},i\right)={\tilde{\Delta }}_{k}\left(t,i\right)z_{k}$, where ${\tilde{\Delta }}_{k}\left(t,i\right)\in R^{m_{k}\times \nu_{k}}$ are arbitrary matrices. We denote as $D_{\ell }$ the set of admissible linear uncertainties. It can be checked that if $△\left(\cdot \right)\in D_{\ell }$, then (16) becomes*

$$\begin{array}{c} |\Delta_{k}\left(\cdot \right)|=\underset{t\in Z_{+}}{\sup}\max_{i\in \Theta }{\left(\lambda_{max}\left[{\tilde{\Delta }}_{k}^{⊤}\left(t,i\right){\tilde{\Delta }}_{k}\left(t,i\right)\right]\right)}^{\frac{1}{2}}. \end{array}$$

*In this special case, (17) recovers the norm of the admissible uncertainties involved in [18].*

Extrapolating the terminology used in [16] to the stochastic case, we shall say that the nominal system (5) is affected by structured uncertainties. The structure of these uncertainties is described by the pairs $(D_{k},C_{k}),1\leq k\leq r$, where $D_{k}={\left\{D_{k}\left(t\right)\right\}}_{t\in Z_{+}},C_{k}={\left\{C_{k}\left(t\right)\right\}}_{t\in Z_{+}}$ with

$$D_{k}\left(t\right)=\left(D_{k}\left(t,1\right),D_{k}\left(t,2\right),\cdots ,D_{k}\left(t,N\right)\right),$$

$$C_{k}\left(t\right)=\left(C_{k}\left(t,1\right),C_{k}\left(t,2\right),\cdots ,C_{k}\left(t,N\right)\right).$$

The nominal system (5) is defined by the sequences $A={\left\{A\left(t\right)\right\}}_{t\in Z_{+}},P={\left\{P_{t}\right\}}_{t\in Z_{+}}$, where A(t)=(A(t,1),A(t,2),⋯,A(t,N)) and where $P_{t}\in R^{N\times N}$ is a stochastic matrix which satisfies the assumption (H3) (i).

Now we are in position to introduce the definition of the stability radius.

**Definition** **3.** *The stability radius of the nominal system (5), or equivalently, the stability radius of the pair (A,P) with respect to the set of structured uncertainties D having the structure described by the pairs $\left(D_{k},C_{k}\right),k\in \underline{r}$, is the number $\rho_{D}\left[A,P|D,C\right]=\inf\{\rho >0|\exists △\left(\cdot \right)\;\mathrm{with}\;∥△\left(\cdot \right)∥\leq \rho$ such that the zero state equilibrium of the corresponding system (1) is not GESMS.*

**Remark** **5.** *When the Definition 3 is restricted to the set of admissible uncertainties $D_{\ell }\subset D$, one obtains the definition of the stability radius $\rho_{\ell }\left[A,P|D,C\right]$ of the nominal system (5) with respect to the linear structured uncertainties involved in [18]. It can be seen that $\rho_{D}\left[A,P|D,C\right]\leq \rho_{\ell }\left[A,P|D,C\right].$*

In [18], the following result was obtained:

**Proposition** **1.** *Assume the following:*

*(a)*  *Assumptions (**H1**)–(**H5**) are fulfilled;*
*(b)*  *The zero solution of the nominal system (5) is SESMS.*

*Under these conditions, the zero solution of the perturbed system (1) is SESMS for every perturbation $△\left(\cdot \right)=\left(\Delta \left(t,1\right),\Delta \left(t,2\right),\ldots ,\Delta \left(t,N\right)\right)\in D_{\ell }$ which satisfies the estimate*

(18) $$\begin{array}{c} ∥△\left(\cdot \right)∥<\tilde{\rho }, \end{array}$$

*where ∥△(·)∥ is defined by (17) and $\tilde{\rho }$ is computed by*

(19) $$\begin{array}{c} {\left(\tilde{\rho }\right)}^{-1}=\underset{t\geq 0}{\sup}\left(\max_{1\leq k\leq r}\left(\max_{i\in \Theta }{\left(\lambda_{\max}\left(D_{k}^{⊤}\left(t,i\right)E_{i}\left(t,\tilde{X}\left(t+1\right)\right)D_{k}\left(t,i\right)\right)\right)}^{1/2}\right)\right), \end{array}$$

*$\tilde{X}\left(t\right)=\left(\tilde{X}\left(t,1\right),\tilde{X}\left(t,2\right),\ldots ,\tilde{X}\left(t,N\right)\right)$ being the unique solution bounded on $Z_{+}$ of the discrete-time backwards affine equation*

(20) $$\begin{array}{c} X\left(t,i\right)=A^{⊤}\left(t,i\right)E_{i}\left(t,X\left(t+1\right)\right)A\left(t,i\right)+\sum_{k=1}^{r}C_{k}^{⊤}\left(t,i\right)C_{k}\left(t,i\right),\;i\in \Theta \end{array}$$

*with*

(21) $$\begin{array}{c} E_{i}\left(t,X\left(t+1\right)\right)=\sum_{j=1}^{N}p_{t}\left(i,j\right)X\left(t+1,j\right). \end{array}$$

In this work, we rely on the scaling technique of perturbations used in [16] to obtain an improved lower bound of the stability radius of the nominal system in (5) with respect to the set D of admissible structured perturbations. To this end, we rewrite the perturbed system (1) in an equivalent form, as follows:

(22a) $$\begin{array}{cc} \; & x\left(t+1\right)=A\left(t,\theta_{t}\right)x\left(t\right)+\sum_{k=1}^{r}w_{k}\left(t\right)\frac{1}{\alpha_{k}\left(\theta_{t}\right)}D_{k}\left(t,\theta_{t}\right)\Delta_{k}^{\alpha }\left(t,z_{k}^{\alpha }\left(t\right),\theta_{t}\right), \end{array}$$

(22b) $$\begin{array}{cc} \; & z_{k}^{\alpha }\left(t\right)=\alpha_{k}\left(\theta_{t}\right)C_{k}\left(t,\theta_{t}\right)x\left(t\right), \end{array}$$

$k\in \underline{r}$, where $\alpha_{k}\left(\theta_{t}\right)\in \left(0,\;\infty \right)$ and

(23) $$\Delta_{k}^{\alpha }\left(t,z_{k}^{\alpha }\left(t\right),\theta_{t}\right)≜\alpha_{k}\left(\theta_{t}\right)\Delta_{k}\left(t,\alpha_{k}^{-1}\left(\theta_{t}\right)z_{k}^{\alpha }\left(t\right),\theta_{t}\right).$$

From (2), (3), and (23), it is apparent that $\Delta_{k}^{\alpha }\left(\cdot \right)\in NL\left(R^{\nu_{k}},R^{m_{k}}\right)$ if $\Delta_{k}\left(\cdot \right)\in NL\left(R^{\nu_{k}},R^{m_{k}}\right)$. Moreover, we may infer from (16) and (23) that $|\Delta_{k}^{\alpha }\left(\cdot \right)|=|\Delta_{k}\left(\cdot \right)|$. Via (17), this allows us to deduce that

(24) $$∥{△}^{\alpha }\left(\cdot \right)∥=∥△\left(\cdot \right)∥$$

for all ${△}^{\alpha }\left(\cdot \right)=\left(\Delta_{1}^{\alpha }\left(\cdot \right),\ldots ,\Delta_{r}^{\alpha }\left(\cdot \right)\right)$. From (23) and (24), we may deduce that $△\left(\cdot \right)\to {△}^{\alpha }\left(\cdot \right):D\to D$ is an isometry for all $\alpha ={\left\{\alpha_{k}\left(i\right)\right\}}_{\left(k,i\right)\in \underline{r}\times \Theta }$, α∈(0,∞).

We consider the discrete-time backward Lyapunov equation (DTBLE) on $S_{n}^{N}$:

(25) $$X\left(t\right)=L_{t}^{*}X\left(t+1\right)+{\left(C^{\alpha }\left(t\right)\right)}^{⊤}C^{\alpha }\left(t\right)$$

where $L_{t}^{*}$ is the linear operator introduced via (11) and ${\left(C^{\alpha }\left(t\right)\right)}^{⊤}C^{\alpha }\left(t\right)=\left(C^{\alpha }{\left(t,1\right)}^{⊤}C^{\alpha }\left(t,1\right),\ldots ,C^{\alpha }{\left(t,N\right)}^{⊤}C^{\alpha }\left(t,N\right)\right)$ with

(26) $$C^{\alpha }{\left(t,i\right)}^{⊤}C^{\alpha }\left(t,i\right)=\sum_{k=1}^{r}\alpha_{k}^{2}\left(i\right)C_{k}^{⊤}\left(t,i\right)C_{k}\left(t,i\right).$$

Applying Theorem 2.5 from [3] in the case of Equation (25), we obtain the following.

**Proposition** **2.** *Assume that:*

*(a)*  *Assumptions (**H2**), (**H3**), and (**H5**) are fulfilled;*
*(b)*  *The zero solution of the nominal system in (5) is SESMS.*

Under these conditions, for any vector of scaling parameters $\alpha ={\left\{\alpha_{k}\left(i\right)\right\}}_{\left(k,i\right)\in \underline{r}\times \Theta }$, the DTBLE (25) has a unique solution $X^{\alpha }\left(\cdot \right)$ which is bounded on $Z_{+}$. This solution is positive semidefinite and has the representation $X^{\alpha }\left(t\right)=\sum_{s=t}^{\infty }R^{*}\left(s,t\right){\left(C^{\alpha }\left(s\right)\right)}^{⊤}C^{\alpha }\left(s\right)$ for all $t\in Z_{+}$. Here, $R^{*}\left(s,t\right)$ is the adjoint operator of the linear evolution operator R(s,t) with respect to the inner product (8). Furthermore, the dependence of the solution $X^{\alpha }\left(\cdot \right)$ with respect to the scaling parameters $\alpha_{k}\left(i\right)$ is described by

(27) $$X^{\alpha }\left(t\right)=\sum_{l=1}^{r}\sum_{i=1}^{N}\alpha_{l}^{2}\left(i\right)X_{li}\left(t\right),$$

*where for each $\left(l,i\right)\in \underline{r}\times \Theta$, we have $X_{li}\left(\cdot \right)=\left(X_{li}\left(\cdot ,1\right),\ldots ,X_{li}\left(\cdot ,N\right)\right)$ as the unique bounded solution of the DTBLE:*

(28a) $$\begin{array}{cc} \; & X_{li}\left(t\right)=L_{t}^{*}X_{li}\left(t+1\right)+C_{li}^{⊤}\left(t\right)C_{li}\left(t\right), \end{array}$$

(28b) $$\begin{array}{cc} \; & C_{li}^{⊤}\left(t\right)C_{li}\left(t\right)=\left(C_{li}^{⊤}\left(t,1\right)C_{li}\left(t,1\right),\ldots ,C_{li}^{⊤}\left(t,N\right)C_{li}\left(t,N\right)\right), \end{array}$$

(28c) $$\begin{array}{cc} \; & C_{li}\left(t,j\right)=\left\{\begin{array}{c} 0\;\mathrm{if}\;j\neq i,\\ C_{l}\left(t,i\right)\;\mathrm{if}\;j=i. \end{array}\right. \end{array}$$

For each $\left(k,\;j\right)\in \underline{r}\times \Theta$ and each vector of scaling parameters $\alpha \in {\left(0,\;\infty \right)}^{\underline{r}\times \Theta }$, we denote

(29) $$H_{kj}\left(t,X^{\alpha }\left(t+1\right)\right)≜D_{k}^{⊤}\left(t,j\right)E_{j}\left(t,X^{\alpha }\left(t+1\right)\right)D_{k}\left(t,j\right),$$

$\forall t\in Z_{+}$, $X^{\alpha }\left(\cdot \right)$ being the unique solution of (25). From (27) and (29), we get

(30) $$H_{kj}\left(t,X^{\alpha }\left(t+1\right)\right)=\sum_{l=1}^{r}\sum_{j=1}^{N}\alpha_{l}^{2}\left(i\right)H_{kj}\left(t,X_{li}\left(t+1\right)\right),$$

$X_{li}\left(\cdot \right)$ being the unique solution of DTLE (28).

We are now in position to prove the following theorem.

**Theorem** **1.** *Assume:*

*(a)*  *Assumptions (**H2**), (**H3**), and (**H5**) are fulfilled;*
*(b)*  *The zero solution of the nominal system in (5) is SESMS.*

*Given σ>0, if there exists $\alpha \in {\left(0,\;\infty \right)}^{\underline{r}\times \Theta }$ such that*

(31) $$\max_{k\in \underline{r}}\;\max_{j\in \Theta }\;\underset{t\in Z_{+}}{\sup}{\left(\frac{\sigma }{\alpha_{k}\left(j\right)}\right)}^{2}∥H_{kj}\left(t,X^{\alpha }\left(t+1\right)\right)∥<1,$$

*then $\rho_{D}\left[A,P\right|\left(D,C\right]\geq \sigma$.*

**Proof.**  Let $△\left(\cdot \right)=\left(\Delta_{1}\left(\cdot \right),\ldots ,\Delta_{r}\left(\cdot \right)\right)\in D$ be arbitrary such that ∥△(·)∥≤σ. We have to show that the system (1) corresponding to this perturbation is GESMS-C. Let $\alpha =\alpha_{k}\left(i\right)$, $\left(k,i\right)\in \underline{r}\times \Theta$ be a vector of scaling parameters satisfying (31). Based on Proposition 2, we deduce that under the considered assumptions, the DTBLE (25) has a unique solution $X^{\alpha }\left(\cdot \right)$, and additionally that this solution is positive semidefinite and bounded on $Z_{+}$. Applying Lemma 3.1 from [3] in the case of the equivalent version (22) of the system in (1), taking $g_{0}\left(t\right)=0$, $g_{k}\left(t\right)=\frac{1}{\alpha_{k}\left(\theta_{t}\right)}D_{k}\left(t,\theta_{t}\right)\Delta_{k}^{\alpha }\left(t,z_{k}^{\alpha }\left(t\right),\theta_{t}\right)$, $k\in \underline{r}$, we obtain

(32) $$\begin{array}{cc} \; & E\left[x^{⊤}\left(t+1\right)X^{\alpha }\left(t+1,\theta_{t+1}\right)x\left(t+1\right)|\theta_{t_{0}}\right]=E\left[x^{⊤}\left(t\right)\left(L_{t}^{*}X^{\alpha }\left(t+1\right)\right)\left(\theta_{t}\right)x\left(t\right)|\theta_{t_{0}}\right]+\\ \; & +\sum_{k=1}^{r}E\left[\frac{1}{\alpha_{k}^{2}\left(\theta_{t}\right)}{\left(\Delta_{k}^{\alpha }\left(t,z_{k}^{\alpha }\left(t\right),\theta_{t}\right)\right)}^{⊤}H_{k\theta_{t}}\left(t,X^{\alpha }\left(t+1\right)\right)\Delta_{k}^{\alpha }\left(t,z_{k}^{\alpha }\left(t\right),\theta_{t}\right)|\theta_{t_{0}}\right] \end{array}$$

$\forall t\geq t_{0}\in Z_{+}$, $x\left(t\right):=x\left(t;t_{0},x_{0}\right)$ being an arbitrary solution of system (22). Using (25), we obtain from (32) that

(33) $$\begin{array}{cc} \; & E\left[x^{⊤}\left(t+1\right)X^{\alpha }\left(t+1,\theta_{t+1}\right)x\left(t+1\right)|\theta_{t_{0}}=i\right]-x_{0}^{⊤}X^{\alpha }\left(t_{0},i\right)x_{0}=\\ \; & \sum_{s=t_{0}}^{t}\sum_{k=1}^{r}\left\{E\left[\frac{1}{\alpha_{k}^{2}\left(\theta_{s}\right)}{\left(\Delta_{k}^{\alpha }\left(s,z_{k}^{\alpha }\left(s\right),\theta_{s}\right)\right)}^{⊤}H_{k\theta_{s}}\left(s,X^{\alpha }\left(s+1\right)\right)\Delta_{k}^{\alpha }\left(s,z_{k}^{\alpha }\left(s\right),\theta_{s}\right)|\theta_{t_{0}}=i\right]\right.\\ \; & \left.-E\left[|z_{k}^{\alpha }\left(s\right){|}^{2}|\theta_{t_{0}}=i\right]\right\}, \end{array}$$

$\forall t\geq t_{0}\in Z_{+},\;i\in \Theta ,\;x_{0}\in R^{n}$. On the other hand, we have

(34) $$\begin{array}{cc} \; & E\left[\frac{1}{\alpha_{k}^{2}\left(\theta_{s}\right)}{\left(\Delta_{k}^{\alpha }\left(s,z_{k}^{\alpha }\left(s\right),\theta_{s}\right)\right)}^{⊤}H_{k\theta_{s}}\left(s,X^{\alpha }\left(s+1\right)\right)\Delta_{k}^{\alpha }\left(s,z_{k}^{\alpha }\left(s\right),\theta_{s}\right)|\theta_{t_{0}}=i\right]\leq \\ \; & \leq \max_{j\in \Theta }\left\{\frac{1}{\alpha_{k}^{2}\left(j\right)}∥H_{kj}\left(s,X^{\alpha }\left(s+1\right)\right)∥E\left[|\Delta_{k}^{\alpha }\left(s,z_{k}^{\alpha }\left(s\right),\theta_{s}\right){|}^{2}|\theta_{t_{0}}=i\right]\right\} \end{array}$$

$\forall t\geq t_{0}\in Z_{+},\;i\in \Theta$.

From (16) and (23), we get

(35) $$E\left[|\Delta_{k}^{\alpha }\left(s,z_{k}^{\alpha }\left(s\right),\theta_{s}\right){|}^{2}|\theta_{t_{0}}=i\right]\leq {\left|\Delta_{k}\left(\cdot \right)\right|}^{2}E\left[|z_{k}^{\alpha }\left(s\right){|}^{2}|\theta_{t_{0}}=i\right].$$

Invoking (17), we deduce from (34) and (35) that

(36) $$\begin{array}{cc} \; & E\left[\frac{1}{\alpha_{k}^{2}\left(\theta_{s}\right)}{\left(\Delta_{k}^{\alpha }\left(s,z_{k}^{\alpha }\left(s\right),\theta_{s}\right)\right)}^{⊤}H_{k\theta_{s}}\left(s,X^{\alpha }\left(s+1\right)\right)\Delta_{k}^{\alpha }\left(s,z_{k}^{\alpha }\left(s\right),\theta_{s}\right)|\theta_{t_{0}}=i\right]\leq \\ \; & \leq \max_{j\in \Theta }\left\{\frac{{∥△\left(\cdot \right)∥}^{2}}{\alpha_{k}^{2}\left(j\right)}∥H_{kj}\left(s,X^{\alpha }\left(s+1\right)\right)∥\right\}E\left[|z_{k}^{\alpha }\left(s\right){|}^{2}|\theta_{t_{0}}=i\right]\\ \; & \leq \max_{j\in \Theta }\left\{{\left(\frac{\sigma }{\alpha_{k}\left(j\right)}\right)}^{2}\underset{s\in Z_{+}}{\sup}∥H_{kj}\left(s,X^{\alpha }\left(s+1\right)\right)∥\right\}. \end{array}$$

For the last inequality, we take into account the assumption that ∥△(·)∥≤σ. From (33) and (36), we obtain

(37) $$\begin{array}{cc} \; & \sum_{s=t_{0}}^{t}\sum_{k=1}^{r}\left(1-\max_{j\in \Theta }\left\{{\left(\frac{\sigma }{\alpha_{k}\left(j\right)}\right)}^{2}\underset{s\in Z_{+}}{\sup}∥H_{kj}\left(s,X^{\alpha }\left(s\right)\right)∥\right\}\right)\cdot E\left[|z_{k}^{\alpha }\left(s\right){|}^{2}|\theta_{t_{0}}=i\right]\\ \; & \leq x_{0}^{⊤}X^{\alpha }\left(t_{0},i\right)x_{0}-E\left[x^{⊤}\left(t+1\right)X^{\alpha }\left(t+1,\theta_{t+1}\right)x\left(t+1\right)|\theta_{t_{0}}=i\right] \end{array}$$

$\forall t\geq t_{0}\in Z_{+},\;i\in \Theta ,\;x_{0}\in R^{n}$. Because $X^{\alpha }\left(\cdot ,i\right)\geq 0$∀i∈Θ and

(38) $$\begin{array}{cc} \delta_{0} & :=1-\max_{k\in \underline{r}}\;\max_{j\in \Theta }\;\underset{s\in Z_{+}}{\sup}\left\{{\left(\frac{\sigma }{\alpha_{k}\left(j\right)}\right)}^{2}∥H_{kj}\left(s,X^{\alpha }\left(s\right)\right)∥\right\}\\ \; & \leq 1-\max_{j\in \Theta }\;\underset{s\in Z_{+}}{\sup}\left\{{\left(\frac{\sigma }{\alpha_{k}\left(j\right)}\right)}^{2}∥H_{kj}\left(s,X^{\alpha }\left(s\right)\right)∥\right\} \end{array}$$

$\forall k\in \underline{r}$, we may infer via (37) that

(39) $$\delta_{0}\sum_{s=t_{0}}^{t}\sum_{k=1}^{r}E\left[|z_{k}^{\alpha }\left(s\right){|}^{2}|\theta_{t_{0}}=i\right]\leq x_{0}^{⊤}X^{\alpha }\left(t_{0},i\right)x_{0}$$

$\forall t\geq t_{0}\in Z_{+}$, i∈Θ, $x_{0}\in R^{n}$. Invoking again the properties of the unique bounded solution $X^{\alpha }\left(\cdot \right)$ of the DTBLE (25) stated in Proposition 2, we deduce that there exists ξ>0 not depending upon $\left(t_{0},i\right)\in Z_{+}\times \Theta$ such that $0\leq X^{\alpha }\left(t_{0},i\right)\leq \xi I_{n}$. In addition, (31) yields $\delta_{0}>0$. Thus, (39) leads to

(40) $$\sum_{k=1}^{r}\sum_{s=t_{0}}^{\infty }E[|z_{k}^{\alpha }{\left(s\right)|}^{2}|\theta_{t_{0}}=i]\leq \frac{\xi }{\delta_{0}}{\left|x_{0}\right|}^{2}$$

$\forall \left(t_{0},i\right)\in Z_{+}\times \Theta$, $x_{0}\in R^{n}$.

Thus, we have shown that $z_{k}^{\alpha }\left(\cdot \right)\in L_{\tilde{H}}^{2}\left(R_{+},R^{\nu_{k}}\right)$ for all $k\in \underline{r}$. Further, (16), (17), and (40) allow us to obtain

(41) $$\begin{array}{cc} \sum_{s=t_{0}}^{\infty }E\left[|\Delta_{k}^{\alpha }\left(s,z_{k}^{\alpha }\left(s\right),\theta_{s}\right){|}^{2}|\theta_{t_{0}}=i\right] & \leq {∥△\left(\cdot \right)∥}^{2}\sum_{s=t_{0}}^{\infty }E[|z_{k}^{\alpha }{\left(s\right)|}^{2}\left|\theta_{t_{0}}=i\right]\\ \; & \leq {∥△\left(\cdot \right)∥}^{2}\xi \delta_{0}^{-1}{\left|x_{0}\right|}^{2} \end{array}$$

$\forall \left(t_{0},i\right)\in Z_{+}\times \Theta$.

Applying Corollary 3.9 (i) from [3], we obtain via (41) that there exists c>0 not depending upon $t_{0},i,x_{0}$ such that $\sum_{t=t_{0}}^{\infty }E\left[|x\left(t;t_{0},x_{0}\right){|}^{2}|\theta_{t_{0}}=i\right]\leq c{\left|x_{0}\right|}^{2}$, $\forall \left(t_{0},i\right)\in Z_{+}\times \Theta$, $x_{0}\in R^{n}$. Thus, we have shown that system (1) is GMSS-C for all △(·)∈D with ∥△(·)∥≤σ. Finally, by applying implication (ii)→(i) from Theorem 3.2 in [17], we conclude that the perturbed system (1) is GESMS-C for any arbitrary △(·)∈D satisfying ∥△(·)∥≤σ. Thus, the proof is complete. □

In addition to the DTBLE (25), we may consider a discrete-time backward Lyapunov inequality (DTBLI) of the following form:

(42) $$-Y\left(t\right)+L_{t}^{*}Y\left(t+1\right)+{\left(C^{\alpha }\left(t\right)\right)}^{⊤}C^{\alpha }\left(t\right)≺≺0$$

$t\in Z_{+}$. Regarding the properties of the solutions of a DTBLI (42), we recall the following lemma.

**Lemma** **1.** *Under assumptions (**H3**)(ii) and (**H5**), the following hold:*

*(i)*  *The nominal system (5) is SESMS if and only if the DTBLI (42) has at least a bounded solution $Y^{\alpha }\left(\cdot \right)$ which is uniformly positive, that is, $Y^{\alpha }\left(t\right)≻≻0$, $t\in Z_{+}$;*
*(ii)*  *If the nominal system (5) is SESMS, then any solution $Y^{\alpha }\left(\cdot \right)$ bounded on $Z_{+}$ of the DTBLI (42) satisfies $Y^{\alpha }\left(t\right)≻≻X^{\alpha }\left(t\right)$, $t\in Z_{+}$, where $X^{\alpha }\left(\cdot \right)$ is the unique bounded solution of the DTBLE (25).*

The next result provides a lower bound for the stability radius in terms of a solution of the DTBLI (42).

**Corollary** **1.** *(a) Assume that the assumptions from Theorem 1 are fulfilled. Given σ>0, there exists a vector of scaling parameters $\alpha \in {\left(0,\infty \right)}^{\underline{r}\times \Theta }$ and a bounded on $Z_{+}$ solution $Y^{\alpha }\left(\cdot \right)$ of the corresponding DTBLI (42) satisfying the condition*

(43) $$\max_{k\in \underline{r}}\;\max_{j\in \Theta }\;\underset{t\in Z_{+}}{\sup}\left\{{\left(\frac{\sigma }{\alpha_{k}\left(j\right)}\right)}^{2}∥H_{kj}\left(t,Y^{\alpha }\left(t+1\right)\right)∥\right\}<1,$$

*where $H_{kj}\left(t,Y^{\alpha }\left(t+1\right)\right)$ is computed as in (30) for $X^{\alpha }\left(t+1\right)$ replaced by $Y^{\alpha }\left(t+1\right)$; then,*

(44) $$\rho_{D}\left[A,P|\left(D,C\right)\right]\geq \sigma .$$

*(b)*  *Assume that assumptions (**H1**)–(**H3**) and (**H5**) are fulfilled. If there exists a vector of scaling parameters $\alpha \in {\left(0,\infty \right)}^{\underline{r}\times \Theta }$ and a solution $Y^{\alpha }\left(\cdot \right)$ bounded on $Z_{+}$ of the corresponding DTBLI (42) that satisfies (43), then the nominal system (5) is SESMS and the stability radius satisfies (44).*

**Proof.**  (a) Based on Lemma 1 (ii), we find that (31) holds if (43) is true, as the spectral norm on the space of symmetric matrices is monotone with respect to the convex cone of the positive semidefinite matrices. The conclusion follows by applying Theorem 1.

(b) The fact that the nominal system in (5) is SESMS follows from Lemma 1 (i). The rest of the proof is similar to the one from (a).

□

The next result provides the best lower bound of the stability radius which may be obtained via the scaling technique.

**Theorem** **2.** *Assume that the assumptions from Theorem 1 are fulfilled. Then:*

(45a) $$\begin{array}{cc} \; & \rho_{D}\left[A,P\right|\left(D,C\right]\geq \hat{\mu }, \end{array}$$

(45b) $$\begin{array}{cc} \; & \hat{\mu }=\underset{\alpha \in {\left(0,\infty \right)}^{\underline{r}\times \Theta }}{\sup}{\left[\max_{k\in \underline{r}}\;\max_{j\in \Theta }\;\underset{s\in Z_{+}}{\sup}\left\{\alpha_{k}^{-2}\left(j\right)∥H_{kj}\left(s,X^{\alpha }\left(s+1\right)\right)∥\right\}\right]}^{-\frac{1}{2}}, \end{array}$$

*$X^{\alpha }\left(\cdot \right)$ being the unique bounded solution of the DTBLE (25) corresponding to the vector of scaling parameter α.*

**Proof.**  By contrast, assuming that (45) is not true, this means that $\rho_{D}\left[A,P\right|\left(D,C\right]<\hat{\mu }$. Let $\sigma \in \left(\rho_{D}\left[A,P|D,C\right],\hat{\mu }\right)$ be arbitrary but fixed. From (45b), we deduce that there exists $\hat{\alpha }={\left\{{\hat{\alpha }}_{k}\left(i\right)\right\}}_{\left(k,i\right)\in \underline{r}\times \Theta }$ from (0,∞) such that $\sigma <{\left[\max_{k\in \underline{r}}\;\max_{j\in \Theta }\;\underset{s\in Z_{+}}{\sup}\left\{{\hat{\alpha }}_{k}^{-2}\left(j\right)∥H_{kj}\left(s,X^{\hat{\alpha }}\left(s+1\right)\right)∥\right\}\right]}^{-\frac{1}{2}}$. This is equivalent to

(46) $$\max_{k\in \underline{r}}\;\max_{j\in \Theta }\;\underset{s\in Z_{+}}{\sup}\left\{{\left(\frac{\sigma }{{\hat{\alpha }}_{k}\left(j\right)}\right)}^{2}∥H_{kj}\left(s,X^{\hat{\alpha }}\left(s+1\right)\right)∥\right\}<1.$$

Applying Theorem 1, we conclude via (46) that $\rho_{D}\left[A,P|D,C\right]\geq \sigma$. However, this is in contradiction with the choice of σ from above. Hence, (46) holds. This completes the proof. □

Adapting the reasoning from [16] to the stochastic framework considered in this work, in the next section we show that in the time-invariant context, the inequality (45a) becomes an equality.

## 4. The Time-Invariant Case

In this section, we assume that system (1) satisfies the following assumption:

**(H6)** 
  (a) A(t,i)=A(i), $D_{k}\left(t,i\right)=D_{k}\left(i\right)$, $C_{k}\left(t,i\right)=C_{k}\left(i\right)$, 1≤k≤r, i∈Θ, $P_{t}=P$, $t\in Z_{+}$;
  (b) The set D of admissible uncertainties is the same as in the previous sections.

Hence, the definition of the stability radius remains unchanged.

**Remark** **6.** *(a) In the special case considered in this section, the nominal system (5) becomes*

(47) $$x\left(t+1\right)=A\left(\theta_{t}\right)x\left(t\right),\;t\in Z_{+}.$$

*According to Theorem 3.10 from [3], in the special case of the period θ=1, the three kinds of exponential stability in the mean square introduced in Section 2 become equivalent in the case of the nominal system (47).*

*(b)*  *The Lyapunov operator (10) and its adjoint become*
(48a) $$\begin{array}{cc} \; & \left(LS\right)\left(i\right)=\sum_{j\in \Theta }p\left(j,i\right)A\left(j\right)S\left(j\right)A^{⊤}\left(j\right), \end{array}$$
(48b) $$\begin{array}{cc} \; & \left(L^{*}S\right)\left(i\right)=\sum_{j\in \Theta }p\left(i,j\right)A^{⊤}\left(i\right)S\left(j\right)A\left(i\right), \end{array}$$
*i∈Θ, $S=\left(S\left(1\right),\ldots ,S\left(N\right)\right)\in S_{n}^{N}$.*
*(c)*  *Applying Theorem 2.5 (iii) from [3] in the case of the DTBLE (25), we deduce that if the zero solution of the nominal system (47) is ESMS, then for each vector of scaling parameters $\alpha \in {\left(0,\infty \right)}^{\underline{r}\times \Theta }$, the DTBLE (25) has a unique bounded solution and that solution is constant. This is the unique solution of the discrete-time algebraic Lyapunov equation DTALE:*
(49) $$X\left(\alpha \right)=L^{*}X\left(\alpha \right)+C^{⊤}\left(\alpha \right)C\left(\alpha \right)$$
  where $L^{*}$ is the linear operator defined in (48b) and where
(50) $$\left[C^{⊤}\left(\alpha \right)C\left(\alpha \right)\right]\left(i\right)=\sum_{k=1}^{r}\alpha_{k}^{2}\left(i\right)C_{k}^{⊤}\left(i\right)C_{k}\left(i\right),\;i\in \Theta .$$
*(d)*  *When the nominal system (47) is ESMS, the dependence of the solution X(α) of the DTALE (49) with respect to the scaling parameters $\alpha_{k}\left(i\right)$ is provided by*
(51) $$X\left(\alpha \right)=\sum_{l=1}^{r}\sum_{i=1}^{N}\alpha_{l}^{2}\left(i\right)X_{li},$$
  *where $X_{li}$ is the unique solution of the DTALE:*
(52a) $$\begin{array}{cc} \; & X_{li}=L^{*}X_{li}+C_{li}^{⊤}C_{li} \end{array}$$
(52b) $$\begin{array}{cc} \; & C_{li}=\left(C_{li}\left(1\right),\ldots ,C_{li}\left(N\right)\right) \end{array}$$
(52c) $$\begin{array}{cc} \; & C_{li}\left(j\right)=\left\{\begin{array}{c} 0\;\mathrm{if}\;j\neq i\\ C_{l}\left(i\right)\;\mathrm{if}\;j=i. \end{array}\right. \end{array}$$
*(e)*  *The time-invariant version of (29) is*
(53) $$H_{kj}\left(X\left(\alpha \right)\right)=D_{k}^{⊤}\left(j\right)\left(\sum_{i=1}^{N}p\left(j,i\right)X\left(\alpha ,i\right)\right)D_{k}\left(j\right)$$
*$\forall \left(k,j\right)\in \underline{r}\times \Theta$, X(α)=(X(α,1),…,X(α,N)) being the unique solution of (49). Combining (51) and (53), we obtain*
(54) $$H_{kj}\left(X\left(\alpha \right)\right)=\sum_{l=1}^{r}\sum_{i=1}^{N}\alpha_{k}^{2}\left(i\right)H_{kj}\left(X_{li}\right),\;\forall \left(k,j\right)\in \underline{r}\times \Theta .$$

Let $I\subset \underline{r}\times \Theta$ be a given subset of indices. We denote as $\alpha^{I}={\left\{\alpha_{k}\left(j\right)\right\}}_{\left(k,j\right)\in \underline{r}\times \Theta }$ an arbitrary element from ${\left[0,\infty \right)}^{\underline{r}\times \Theta }$ with the properties $\alpha_{k}\left(j\right)>0$ if (k,j)∈I and $\alpha_{k}\left(j\right)=0$ if (k,j) are not in I.

Let $X(\alpha^{I})$ be the unique solution of the DTALE:

(55) $$X\left(\alpha^{I}\right)=L^{*}X\left(\alpha^{I}\right)+C^{⊤}\left(\alpha^{I}\right)C\left(\alpha^{I}\right)$$

where $L^{*}$ is the Lyapunov-type operator defined in (48b) and $C^{⊤}\left(\alpha^{I}\right)C\left(\alpha^{I}\right)\in S_{n}^{N}$, with its i-th block component defined by

(56) $$\left(C^{⊤}\left(\alpha^{I}\right)C\left(\alpha^{I}\right)\right)\left(i\right)=\sum_{k=1}^{r}\alpha_{k}^{2}\left(i\right)C_{k}^{⊤}\left(i\right)C_{k}\left(i\right),$$

where $\alpha_{k}\left(i\right)$ stands for the components of the vector $\alpha^{I}$. If the zero solution of the nominal system in (47) is ESMS, then for each $\alpha^{I}\in {\left[0,\infty \right)}^{\underline{r}\times \Theta }$, the DTALE (55) and (56) has a unique solution $X\left(\alpha^{I}\right)\in S_{n}^{N+}$.

The following technical result is involved in the proof of the main result of this section.

**Lemma** **2.** *Assume the following:*

*(a)*  *Assumptions (* ***H1*** *)–(* ***H3*** *) and (* ***H6*** *) are fulfilled;*
*(b)*  *The nominal system in (47) is ESMS.*

*We make the following convention of calculation: $\frac{1}{\alpha_{k}\left(j\right)}=0$ if $\alpha_{k}\left(j\right)=0$.*

*Let $x(t,x_{0})$, $t\in Z_{+}$ be the solution of the initial value problem (IVP):*

(57) $$x\left(t+1\right)=A\left(\theta_{t}\right)x\left(t\right)+\sum_{k=1}^{r}\frac{w_{k}\left(t\right)}{\alpha_{k}\left(\theta_{t}\right)}D_{k}\left(\theta_{t}\right)v_{k}\left(t\right);\;t\geq 0,\;x\left(0\right)=x_{0}\in R^{n}$$

*and ${\left\{v_{k}\left(t\right)\right\}}_{t\geq 0}\in l_{\tilde{H}}^{2}\left(Z_{+},R^{m_{k}}\right)$, 1≤k≤r.*

*If $z^{\alpha^{I}}\left(t,x_{0}\right)=\left({\left(z_{1}^{\alpha^{I}}\left(t,x_{0}\right)\right)}^{⊤},{\left(z_{2}^{\alpha^{I}}\left(t,x_{0}\right)\right)}^{⊤},\cdots ,{\left(z_{r}^{\alpha^{I}}\left(t,x_{0}\right)\right)}^{⊤}\right)*⊤$ with*

(58) $$z_{k}^{\alpha^{I}}\left(t,x_{0}\right)=\alpha_{k}\left(\theta_{t}\right)C_{k}\left(\theta_{t}\right)x\left(t,x_{0}\right)$$

*$\alpha_{k}\left(\theta_{t}\right)$ being the components of the scaling vector $\alpha^{I}$ evaluated for $\theta_{t}=j$, then we have*

(59) $$\begin{array}{c} \sum_{t=0}^{\infty }E\left[|z^{\alpha^{I}}\left(t,x_{0}\right){|}^{2}|\theta_{0}=i\right]=x_{0}^{⊤}X\left(\alpha^{I},i\right)x_{0}+\sum_{k=1}^{r}\sum_{t=0}^{\infty }E\left[\frac{1}{\alpha_{k}^{2}\left(\theta_{t}\right)}v_{k}^{⊤}\left(t\right)H_{k\theta_{t}}\left(X\left(\alpha^{I}\right)\right)v_{k}\left(t\right)|\theta_{0}=i\right] \end{array}$$

*∀i∈Θ, $x_{0}\in R^{n}$.*

**Proof.**  Applying Lemma 3.1 from [3] in the case of system (57) and the function $V\left(x\left(t\right),i\right)=x^{⊤}\left(t\right)X\left(\alpha^{I},i\right)x\left(t\right)$, where $X\left(\alpha^{I}\right)=\left(X\left(\alpha^{I},1\right),\ldots ,X\left(\alpha^{I},N\right)\right)$ is the unique solution for the DTALE (55), we obtain

$$\begin{array}{cc} E\left[x^{⊤}\left(t+1,x_{0}\right)X\left(\alpha^{I},\theta_{t+1}\right)x\left(t+1,x_{0}\right)|\theta_{0}\right] & =E\left[x^{⊤}\left(t,x_{0}\right)\left(L^{*}X\left(\alpha^{I}\right)\right)x\left(t,x_{0}\right)|\theta_{0}\right]\\ \; & +\sum_{k=1}^{r}E\left[\frac{1}{\alpha_{k}^{2}\left(\theta_{t}\right)}v_{k}^{⊤}\left(t\right)H_{k\theta_{t}}\left(X\left(\alpha^{I}\right)\right)v_{k}\left(t\right)|\theta_{0}\right]. \end{array}$$

Further, employing (49) and summing from t=0 to t=τ, we get

(60) $$\begin{array}{cc} \sum_{t=0}^{\tau }E\left[|z^{\alpha^{I}}\left(t,x_{0}\right){|}^{2}|\theta_{0}=i\right] & =x_{0}^{⊤}X\left(\alpha^{I},i\right)x_{0}-E\left[x^{⊤}\left(\tau +1,x_{0}\right)X\left(\alpha^{I},\theta_{\tau +1}\right)x\left(\tau +1,x_{0}\right)|\theta_{0}=i\right]\\ \; & +\sum_{k=1}^{r}\sum_{t=0}^{\tau }E\left[\frac{1}{\alpha_{k}^{2}\left(\theta_{t}\right)}v_{k}^{⊤}\left(t\right)H_{k\theta_{t}}\left(X\left(\alpha^{I}\right)\right)v_{k}\left(t\right)|\theta_{0}=i\right]. \end{array}$$

Because ${\left\{v_{k}\left(t\right)\right\}}_{t\in Z_{+}}$ lies in $l_{\tilde{H}}^{2}\left(Z_{+},R^{m_{k}}\right)$, we deduce via Corollary 3.9 (i) from [3] that the series.

$\sum_{t=0}^{\infty }E\left[|x\left(t,x_{0}\right){|}^{2}|\theta_{0}=i\right]$ are convergent for all i∈Θ and $x_{0}\in R^{n}$.

Hence, $\lim_{\tau \to \infty }E\left[x^{⊤}\left(\tau +1,x_{0}\right)X\left(\alpha^{I},\theta_{\tau +1}\right)x\left(\tau +1,x_{0}\right)|\theta_{0}=i\right]=0$

∀i∈Θ, $x_{0}\in R^{n}$. Taking the limit for τ→∞ in (60), we obtain (59). Thus, the proof is complete. □

We are now in position to provide the main result of this section.

**Theorem** **3.** *Assume the following:*

*(a)*  *Assumptions (* ***H1*** *)–(* ***H3*** *) and (* ***H6*** *) are fulfilled;*
*(b)*  *The zero solution of the nominal system in (47) is ESMS.*

*Under these conditions, the stability radius of the nominal system (47) with respect to the set D of the structured uncertainties with the structure described by $\left(D_{k},C_{k}\right)$$k\in \underline{r}$ is provided by*

(61) $$\rho_{D}\left[A,P|{\left(D_{k},C_{k}\right)}_{k\in \underline{r}}\right]=\underset{\alpha \in {\left(0,\infty \right)}^{\underline{r}\times \Theta }}{\sup}{\left[\max_{k\in \underline{r}}\;\max_{j\in \Theta }\left(\alpha_{k}^{-2}\left(j\right)∥H_{kj}\left(X\left(\alpha \right)\right)∥\right)\right]}^{-\frac{1}{2}}.$$

**Proof.**  Let

(62) $$\begin{array}{c} \tilde{\mu }≜\underset{\alpha \in {\left(0,\infty \right)}^{\underline{r}\times \Theta }}{\inf}\;\max_{k\in \underline{r}}\;\max_{j\in \Theta }\left(\alpha_{k}^{-2}\left(j\right)∥H_{kj}\left(X\left(\alpha \right)\right)∥\right)=\underset{\alpha \in {\left(0,\infty \right)}^{\underline{r}\times \Theta }}{\inf}\;\max_{\left(k,j\right)\in \underline{r}\times \Theta }∥\alpha_{k}^{-2}\left(j\right)H_{kj}\left(X\left(\alpha \right)\right)∥. \end{array}$$

With this notation, (61) becomes

(63) $$\rho_{D}\left[A,P|{\left(D_{k},C_{k}\right)}_{k\in \underline{r}}\right]={\tilde{\mu }}^{-\frac{1}{2}}.$$

From Theorem 2, we know that

(64) $$\rho_{D}\left[A,P|{\left(D_{k},C_{k}\right)}_{k\in \underline{r}}\right]\geq {\tilde{\mu }}^{-\frac{1}{2}}.$$

From (64), it is clear that if $\tilde{\mu }=0$, then $\rho_{D}\left[A,P|{\left(D_{k},C_{k}\right)}_{k\in \underline{r}}\right]=\infty$, which confirms (61) in this case.

In the sequelae, we assume that $\tilde{\mu }>0$. Employing (51), we rewrite (62) in the following form:

(65) $$\tilde{\mu }=\underset{\alpha \in {\left(0,\infty \right)}^{\underline{r}\times \Theta }}{\inf}\;\max_{\left(k,j\right)\in \underline{r}\times \Theta }∥\sum_{l=1}^{r}\sum_{i=1}^{N}{\left(\frac{\alpha_{l}\left(i\right)}{\alpha_{k}\left(j\right)}\right)}^{2}H_{kj}\left(X_{li}\right)∥.$$

It can be seen that the value of $\tilde{\mu }$ is obtained by solving a mixed optimization problem similar to that studied in Section 2 of [16]. Hence, by applying Theorem 2.4 from [16], in the case of the optimization problem (65), we deduce that there exists $I\subset \underline{r}\times \Theta$ and a vector of scaling parameters ${\hat{\alpha }}^{I}\in {\left(0,\infty \right)}^{I}$ with the following property that

(66) $$\tilde{\mu }=∥\sum_{(l,i)\in I}{\left(\frac{{\hat{\alpha }}_{l}^{I}\left(i\right)}{{\hat{\alpha }}_{k}^{I}\left(j\right)}\right)}^{2}H_{kj}\left(X_{li}\right)∥=∥\frac{1}{{\left({\hat{\alpha }}_{kj}^{I}\right)}^{2}}H_{kj}\left(X\left({\hat{\alpha }}^{I}\right)\right)∥$$

∀(k,j)∈I, where $X(\alpha^{I})$ is the solution of the DTALE (55) corresponding to the parameter

(67) $$\alpha_{l}^{I}\left(i\right)=\left\{\begin{array}{c} {\hat{\alpha }}_{l}^{I}\left(i\right)\;\mathrm{if}\;\left(l,i\right)\in I\\ 0\;\;\mathrm{if}\;\left(l,i\right)\in \underline{r}\times \Theta -I. \end{array}\right.$$

For each (k,j)∈I we choose ${\tilde{v}}_{kj}\in R^{m_{k}}$ with the properties $∥{\tilde{v}}_{kj}∥=1$ and

(68) $$\frac{1}{{\hat{\alpha }}_{k}^{2}\left(j\right)}{\tilde{v}}_{kj}^{⊤}H_{kj}\left(X\left(\alpha^{I}\right)\right){\tilde{v}}_{kj}=∥\frac{1}{{\hat{\alpha }}_{k}^{2}\left(j\right)}H_{kj}\left(X\left(\alpha^{I}\right)\right)∥=\tilde{\mu }.$$

Let us consider $△\left(\cdot \right)=\left(\Delta_{1}\left(\cdot \right),\ldots ,\Delta_{r}\left(\cdot \right)\right)$, defined as follows:

(69) $$\Delta_{k}\left(t,z_{k},j\right)=\left\{\begin{array}{c} {\tilde{\mu }}^{-\frac{1}{2}}\left|z_{k}\right|{\tilde{v}}_{kj}\;\mathrm{if}\;\left(k,j\right)\in I,\\ 0\;\;\mathrm{if}\;\left(k,j\right)\in \underline{r}\times \Theta -I. \end{array}\right.$$

From (16), (17), and (69) we get $∥△(\cdot )∥={\tilde{\mu }}^{-\frac{1}{2}}$. We show that the perturbed system (1) associated with the uncertainty (69) is not GESMS-C. Assuming by contrast that system (1) is GESMS-C, this means that there exist β≥1, γ∈(0,1) such that the solutions $x(t;x_{0})$ of the considered system satisfy

$$E\left[|x\left(t;x_{0}\right){|}^{2}|\theta_{0}=i\right]\leq \beta \gamma^{t}{\left|x_{0}\right|}^{2}$$

$\forall t\in Z_{+}$, $x_{0}\in R^{n}$, i∈Θ. This leads to

(70) $$\sum_{t=0}^{\infty }E\left[|x\left(t;x_{0}\right){|}^{2}|\theta_{0}=i\right]\leq c{\left|x_{0}\right|}^{2}$$

$\forall x_{0}\in R^{n}$, i∈Θ where $c=\beta {\left(1-\gamma \right)}^{-1}$.

We rewrite the system in (1) corresponding to (69) in the following form:

(71) $$x\left(t+1\right)=A\left(\theta_{t}\right)x\left(t\right)+\sum_{k=1}^{r}w_{k}\left(t\right)\frac{1}{\alpha_{k}^{I}\left(\theta_{t}\right)}D_{k}\left(\theta_{t}\right)v_{k}^{\alpha^{I}}\left(t\right)$$

where $\alpha_{k}^{I}\left(\theta_{t}\right)$ are defined as in (67) for $\theta_{t}=j$ and $v_{k}^{\alpha^{I}}\left(t\right)=\Delta_{k}\left(t,z_{k}^{\alpha^{I}}\left(t\right),\theta_{t}\right)$.

Based on (69), we infer that

(72) $$v_{k}^{\alpha^{I}}\left(t\right)=\left\{\begin{array}{c} {\tilde{\mu }}^{-\frac{1}{2}}\left|z_{k}^{\alpha^{I}}\left(t\right)\right|{\tilde{v}}_{k\theta_{t}}\;\mathrm{if}\;\left(k,\theta_{t}\right)\in I\\ 0\;\;\mathrm{if}\;\left(k,\theta_{t}\right)\in \underline{r}\times \Theta -I \end{array}\right.$$

$t\in Z_{+}$, $k\in \underline{r}$.

Here, $z_{k}^{\alpha^{I}}\left(t\right)$ are computed as in (58) for the scaling parameters $\alpha_{k}^{I}\left(j\right)$ defined in (67) and the solution $x(t,x_{0})$ of (71). From (70), we deduce that ${\left\{z_{k}^{\alpha^{I}}\left(t\right)\right\}}_{t\in Z_{+}}$ is in $\ell_{\tilde{H}}^{2}\left(Z_{+},R^{m_{k}}\right)$, $k\in \underline{r}$. This allows us to deduce that ${\left\{v_{k}^{\alpha^{I}}\left(t\right)\right\}}_{t\in Z_{+}}$ lies in $\ell_{\tilde{H}}^{2}\left(Z_{+},R^{m_{k}}\right)$. Applying Lemma 2, we obtain

(73) $$\begin{array}{c} \sum_{t=0}^{\infty }E\left[|z^{\alpha^{I}}\left(t,x_{0}\right){|}^{2}\right]=\sum_{i=1}^{N}\pi_{0}\left(i\right)x_{0}^{⊤}X\left(\alpha^{I},i\right)x_{0}+\sum_{t=0}^{\infty }\sum_{k=1}^{r}E\left[\varphi_{k}\left(t\right)\right], \end{array}$$

where we have denoted

(74) $$\varphi_{k}\left(t\right)=\frac{1}{{\left(\alpha_{k}^{I}\left(\theta_{t}\right)\right)}^{2}}{\left(v_{k}^{\alpha^{I}}\left(t\right)\right)}^{⊤}H_{k\theta_{t}}\left(X\left(\alpha^{I}\right)\right)v_{k}^{\alpha^{I}}\left(t\right).$$

Now, we have

$$E\left[\varphi_{k}\left(t\right)\right]=\int_{\Omega_{1}^{k}}\varphi_{k}\left(t\right)\left(\omega \right)P\left(d\omega \right)+\int_{\Omega_{2}^{k}}\varphi_{k}\left(t\right)\left(\omega \right)P\left(d\omega \right),$$

where $\Omega_{1}^{k}=\left\{\omega \in \Omega |(k,\theta_{t})\in I\right\}$ and $\Omega_{2}^{k}=\Omega -\Omega_{1}^{k}$.

Based on (67), (68), (72), and (74), we get

(75a) $$\begin{array}{cc} \; & \int_{\Omega_{1}^{k}}\varphi_{k}\left(t\right)\left(\omega \right)P\left(d\omega \right)=\int_{\Omega_{1}^{k}}{\left|z_{k}^{\alpha^{I}}\left(t,x_{0}\right)\left(\omega \right)\right|}^{2}P\left(d\omega \right), \end{array}$$

(75b) $$\begin{array}{cc} \; & \int_{\Omega_{2}^{k}}\varphi_{k}\left(t\right)\left(\omega \right)P\left(d\omega \right)=0. \end{array}$$

On the other hand, (58) and (67) yield

$$\int_{\Omega_{2}^{k}}{\left|z_{k}^{\alpha^{I}}\left(t,x_{0}\right)\left(\omega \right)\right|}^{2}P\left(d\omega \right)=0.$$

This equality together with (75) allows us to conclude that

(76) $$E\left[\varphi_{k}\left(t\right)\right]=E[|z_{k}^{\alpha^{I}}\left(t,x_{0}\right)\left(\omega \right){|}^{2}],\;\forall k\in \underline{r},x_{0}\in R^{n}.$$

Plugging (76) into (73), we deduce that

$$\sum_{i=1}^{N}\pi_{0}\left(i\right)x_{0}^{⊤}X\left(\alpha^{I},i\right)x_{0}=0,\;\forall x_{0}\in R^{n}.$$

This is equivalent to $X(\alpha^{I})=0$, as $\pi_{0}\left(i\right)>0$, i∈Θ according to (**H3**)(iii). Thus, we have obtained via (66) that $\tilde{\mu }=0$. This contradicts our supposition that $\tilde{\mu }>0$. Hence, the system in (1) corresponding to the uncertainty in (69) is not GESMS-C. Thus, we have shown that (63) is true. This complete the proof. □

The next result is often used in designing a robust stabilizing control with a prescribed level of robustness.

**Proposition** **3.** *Assume that assumptions (**H1**)–(**H3**) and (**H6**) are fulfilled. For a given scalar σ>0, the following are equivalent:*

*(i)*  *The nominal system (47) is ESMS and $\rho_{D}\left[A,P|{\left(D_{k},C_{k}\right)}_{k\in \underline{r}}\right]>\sigma$;*
*(ii)*  *There exist positive definite matrices Y(i) and scalars $\alpha_{k}\left(i\right)\in \left(0,\infty \right)$, $k\in \underline{r}$, i∈Θ satisfying the following system of inequalities:*
(77a) $$\begin{array}{cc} \; & \sum_{j=1}^{N}A^{⊤}\left(i\right)p_{ij}Y\left(j\right)A\left(i\right)-Y\left(i\right)+\sum_{k=1}^{r}\alpha_{k}^{2}\left(i\right)C_{k}^{⊤}\left(i\right)C_{k}\left(i\right)<0 \end{array}$$
(77b) $$\begin{array}{cc} \; & \alpha_{k}^{2}\left(i\right)I_{m_{k}}-\sigma^{2}D_{k}^{⊤}\left(i\right)\left(\sum_{j=1}^{N}p_{ij}Y\left(j\right)\right)D_{k}\left(i\right)>0 \end{array}$$
*1≤k≤r, 1≤i≤N.*

**Proof.**  We start with the proof of the implication that (i)→ (ii). If (i) holds, then (59) is true. Let $\sigma_{1}\in \left(\sigma ,\rho_{D}\left[A,P|{\left(D_{k},C_{k}\right)}_{k\in \underline{r}}\right]\right)$. From (61), we deduce that there exists $\alpha ={\left(\alpha_{k}\left(j\right)\right)}_{\left(k,j\right)\in \underline{r}\times \Theta }$ in ${\left(0,\infty \right)}^{\underline{r}\times \Theta }$ such that $\sigma_{1}^{2}<\left[\max_{\left(k,j\right)\in \underline{r}\times \Theta }∥\frac{1}{\alpha_{k}^{2}\left(j\right)H_{kj}\left(X\left(\alpha \right)\right)}∥\right]$, where $X\left(\alpha \right)=\left(X(\alpha ,1),\ldots ,X(\alpha ,N)\right)$ is the unique solution of the DTALE (49) corresponding to the vector of scaling parameters α and $H_{kj}\left(X\left(\alpha \right)\right)$ is computed as in (53).

The previous inequality is equivalent to $∥{\left(\frac{\sigma_{1}}{\alpha_{k}\left(j\right)}\right)}^{2}H_{kj}\left(X\left(\alpha \right)\right)∥<1$

$\forall \left(k,j\right)\in \underline{r}\times \Theta$, which leads to ${\left(\frac{\sigma_{1}}{\alpha_{k}\left(j\right)}\right)}^{2}H_{kj}\left(X\left(\alpha \right)\right)\leq I_{m_{k}}$. Hence,

(78) $${\left(\frac{\sigma }{\alpha_{k}\left(j\right)}\right)}^{2}H_{kj}\left(X\left(\alpha \right)\right)\leq I_{m_{k}},$$

because $\sigma <\sigma_{1}$. On the other hand, if the nominal system in (47) is ESMS, then the eigenvalues of the linear operator $L^{*}$ introduced via (48b) are located in the interior of the disk |λ|<1. In this case, the unique solution X(α) of the DTALE (49) has the representation

(79) $$X\left(\alpha \right)=\sum_{t=0}^{\infty }{\left(L^{*}\right)}^{t}\left(C^{⊤}\left(\alpha \right)C\left(\alpha \right)\right).$$

For ϵ>0, let

(80) $$Y\left(\alpha ,\epsilon \right)≜\sum_{t=0}^{\infty }{\left(L^{*}\right)}^{t}\left(C^{⊤}\left(\alpha \right)C\left(\alpha \right)+\epsilon I\right),$$

where $I=\left(I_{n},I_{n},\cdots ,I_{n}\right)\in S_{n}^{N+}$. From (80) and (79), we get

(81) $$∥Y(\alpha ,\epsilon )-X(\alpha )∥\leq \hat{c}\epsilon ,$$

where $\hat{c}>0$ is a constant. From (80), it is apparent that Y(α,ϵ) satisfies

$$Y\left(\alpha ,\epsilon \right)=L^{*}Y\left(\alpha ,\epsilon \right)+C^{⊤}\left(\alpha \right)C\left(\alpha \right)+\epsilon I.$$

The component-wise version of this equation is

$$Y\left(\alpha ,\epsilon \right)\left(i\right)=\sum_{j=1}^{N}p_{ij}A^{⊤}\left(i\right)Y\left(\alpha ,\epsilon \right)\left(j\right)A\left(i\right)+\sum_{k=1}^{r}\alpha_{k}^{2}\left(i\right)C_{k}^{⊤}\left(i\right)C_{k}\left(i\right)+\epsilon I_{n},\;i\in \Theta .$$

Thus, for each ϵ>0, the matrices Y(α,ϵ)(i) are positive definite and solve the system of LMIs in (77a). In addition, from (78) and (81), we can deduce that for small enough ϵ>0, the matrices Y(α,ϵ)(i) solve the LMIs in (77b). Thus, we have shown that (ii) holds if (i) is true.

To prove the converse implication, let us remark that if (77a) is solvable, then the nominal system in (47) is ESMS.

The condition $\sigma <\rho_{D}\left[A,P|{\left(D_{k},C_{k}\right)}_{k\in \underline{r}}\right]$ follows from Corollary 1. This ends the proof. □

## 5. Robust Stabilization by State Feedback

Let us consider the following discrete-time controlled system:

(82a) $$\begin{array}{cc} \; & x\left(t+1\right)=A\left(\theta_{t}\right)x\left(t\right)+\sum_{k=1}^{r}w_{k}\left(t\right)D_{k}\left(\theta_{t}\right)\Delta_{k}\left(t,z_{k}\left(t\right),\theta_{t}\right)+B\left(\theta_{t}\right)u\left(t\right), \end{array}$$

(82b) $$\begin{array}{cc} \; & z_{k}\left(t\right)=C_{k}\left(\theta_{t}\right)x\left(t\right), \end{array}$$

$k\in \underline{r}$, where x(t) are the state parameters and $u\left(t\right)\in R^{n_{u}}$ are the control parameters.

The stochastic processes ${\left\{w\left(t\right)\right\}}_{t\in Z_{+}}$ and ${\left\{\theta \left(t\right)\right\}}_{t\in Z_{+}}$ are the same as in the case of system (1) and satisfy assumptions (**H1**)–(**H3**). In (82), we have A(i), B(i), $C_{k}\left(i\right)$, $D_{k}\left(i\right)$, $k\in \underline{r}$, i∈Θ as the known matrices of appropriate dimensions and $z_{k}\to \Delta_{k}\left(t,z_{k},i\right)$ as arbitrary functions from the set D. The system in (82) can be regarded as a disturbance of the nominal system

(83) $$x\left(t+1\right)=A\left(\theta_{t}\right)x\left(t\right)+B\left(\theta_{t}\right)u\left(t\right).$$

For robust stabilization of the nominal system in (83) with respect to the structured uncertainties from D having the structure described by ${\left(D_{k},C_{k}\right)}_{k\in \underline{r}}$, we can use controls of the following form:

(84) $$u\left(t\right)=F\left(\theta_{t}\right)x\left(t\right)$$

where $F\left(i\right)\in R^{n_{u}\times n}$. The system obtained by coupling a control of type (84) to the system in (82) is as follows:

(85a) $$\begin{array}{cc} \; & x\left(t+1\right)=\left(A\left(\theta_{t}\right)+B\left(\theta_{t}\right)F\left(\theta_{t}\right)\right)x\left(t\right)+\sum_{k=1}^{r}w_{k}\left(t\right)D_{k}\left(\theta_{t}\right)\Delta_{k}\left(t,z_{k}\left(t\right),\theta_{t}\right), \end{array}$$

(85b) $$\begin{array}{cc} \; & z_{k}\left(t\right)=C_{k}\left(\theta_{t}\right)x\left(t\right), \end{array}$$

$k\in \underline{r}$. The system in (85) can be regarded as a disturbance of the system

(86) $$x\left(t+1\right)=\left(A\left(\theta_{t}\right)+B\left(\theta_{t}\right)F\left(\theta_{t}\right)\right)x\left(t\right).$$

It can be seen that the system in (86) is the closed-loop system obtained by coupling a control of type (84) to the nominal system in (83). For this reason, in the sequelae, we refer to the system in (86) as *the closed-loop nominal system*.

Our aim is to find necessary and sufficient conditions which guarantee the existence of the control of type (84) which stabilizes the closed-loop nominal system in (86), and additionally its stability radius with respect to the structured uncertainties from D:

(87) $$\rho_{D}\left[A+BF,P|{\left(D_{k},C_{k}\right)}_{k\in \underline{r}}\right]>\sigma$$

for a prefixed level of robustness σ. A first answer to this problem is provided in the following theorem.

**Theorem** **4.** *Assume the following:*

*(a)*  *Assumptions (**H1**)–(**H3**) and (**H6**) are fulfilled;*
*(b)*  *$\mathrm{rank}\;B\left(i\right)=n_{u}$,i∈Θ.*

*For a given level of robustness σ>0, the following are equivalent:*

*(i)*  *There exists a control in a state feedback form (84) which stabilizes the nominal system in (83) such that the stability radius of the closed-loop nominal system satisfies (87);*
*(ii)*  *There exist scalars $\alpha_{k}\left(i\right)\in \left(0,\infty \right)$, $\left(k,i\right)\in \underline{r}\times \Theta$ and positive definite matrices $X\left(i\right)\in S_{n}$, i∈Θ satisfying the inequalities*
(88a) $$\begin{array}{cc} \; & A^{⊤}\left(i\right)E_{i}\left(X\right)A\left(i\right)-X\left(i\right)+\sum_{k=1}^{r}\alpha_{k}^{2}\left(i\right)C_{k}^{⊤}\left(i\right)C_{k}\left(i\right)-A^{⊤}\left(i\right)E_{i}\left(X\right)B\left(i\right){\left(B^{⊤}\left(i\right)E_{i}\left(X\right)B\left(i\right)\right)}^{-1}★<0, \end{array}$$
(88b) $$\begin{array}{cc} \; & I_{m_{k}}-{\left(\frac{\sigma }{\alpha_{k}\left(i\right)}\right)}^{2}D_{k}^{⊤}\left(i\right)E_{i}\left(X\right)D_{k}\left(i\right)>0, \end{array}$$
  *i∈Θ, where*
(89) $$E_{i}\left(X\right)=\sum_{j=1}^{N}p\left(i,j\right)X\left(j\right).$$
  *Moreover, if (88) is solvable, then a control of type (84) which robustly stabilizes the nominal system in (83) while achieving a stability radius which satisfies (87) has a gain matrix provided by*
(90) $$\hat{F}\left(i\right)=-{\left(B^{⊤}\left(i\right)E_{i}\left(X\right)B\left(i\right)\right)}^{-1}B^{⊤}\left(i\right)E_{i}\left(X\right)A\left(i\right),\;i\in \Theta .$$

**Proof.**  (i) ⇒ (ii). Let us assume that there exists a control of type (84) which stabilizes the nominal system in (83) such that the achieved stability radius satisfies (87). Applying the implication (i) ⇒ (ii) from Proposition 3 in the case of systems (85) and (86), we deduce that there exist scalars $\alpha_{k}\left(i\right)>0$, $\left(k,i\right)\in \underline{r}\times \Theta$ and $X=\left(X\left(1\right),\cdots ,X\left(N\right)\right)\in S_{n}^{N}$ such that X(j)>0, j∈Θ, which satisfy the LMIs

(91a) $$\begin{array}{cc} \; & {\left(A\left(i\right)+B\left(i\right)F\left(i\right)\right)}^{⊤}E_{i}\left(X\right)\left(A\left(i\right)+B\left(i\right)F\left(i\right)\right)-X\left(i\right)+\sum_{k=1}^{r}\alpha_{k}^{2}\left(i\right)C_{k}^{⊤}\left(i\right)C_{k}\left(i\right)<0, \end{array}$$

(91b) $$\begin{array}{cc} \; & I_{m_{k}}-{\left(\frac{\sigma }{\alpha_{k}\left(i\right)}\right)}^{2}D_{k}^{⊤}\left(i\right)E_{i}\left(X\right)D_{k}\left(i\right)>0, \end{array}$$

i∈Θ. From assumption (b) and (89), we deduce that

(92) $$B^{⊤}\left(i\right)E_{i}\left(X\right)B\left(i\right)>0,$$

because X(i)>0 and $\sum_{j=1}^{N}p\left(i,j\right)=1$. This allows us to rewrite (91a) in the following form:

(93) $$\begin{array}{cc} \; & A^{⊤}\left(i\right)E_{i}\left(X\right)A\left(i\right)-X\left(i\right)+{\left(F\left(i\right)-\hat{F}\left(i\right)\right)}^{⊤}\left(B^{⊤}\left(i\right)E_{i}\left(X\right)B\left(i\right)\right)\left(F\left(i\right)-\hat{F}\left(i\right)\right),\\ \; & +\sum_{k=1}^{r}\alpha_{k}^{2}\left(i\right)C_{k}^{⊤}\left(i\right)C_{k}\left(i\right)-A^{⊤}\left(i\right)E_{i}\left(X\right)B\left(i\right){\left(B^{⊤}\left(i\right)E_{i}\left(X\right)B\left(i\right)\right)}^{-1}★<0, \end{array}$$

where $\hat{F}\left(i\right)$ is defined in (90). Taking into account (92), we may infer that (88) is solvable if (93) and (91) are solvable. Thus, we have shown that (ii) holds if (i) is true.

In order to prove the converse implication, let us remark that (88a) together with (90) leads to (93), which is equivalent to (91a). The conclusion is obtained by invoking (ii) ⇒ (i) from Proposition 3 in the case of system (86). □

**Remark** **7.** *It is apparent that (88a) cannot be converted in an obvious way into an LMI because of (92). This makes the numerical computation of the gain matrices in (90) difficult, since they are based on a solution of (88). This leads us to replace (88a) with the discrete-time algebraic Riccati equation (DTARE):*

(94) $$\begin{array}{c} X\left(i\right)=A^{⊤}\left(i\right)E_{i}\left(X\right)A\left(i\right)-A^{⊤}\left(i\right)E_{i}\left(X\right)B\left(i\right){\left(B^{⊤}\left(i\right)E_{i}\left(X\right)B\left(i\right)\right)}^{-1}★+\sum_{k=1}^{r}\alpha_{k}^{2}\left(i\right)C_{k}^{⊤}\left(i\right)C_{k}\left(i\right). \end{array}$$

**Definition** **4.** *A solution $\tilde{X}=\left(\tilde{X}\left(1\right),\ldots ,\tilde{X}\left(N\right)\right)$ of the DTARE (94) is called a stabilizing solution if $\det\left(B^{⊤}\left(i\right)E_{i}\left(\tilde{X}\right)B\left(i\right)\right)\neq 0$∀i∈Θ and if the zero solution of the closed-loop system*

(95a) $$\begin{array}{cc} \; & x\left(t+1\right)=\left(A\left(\theta_{t}\right)+B\left(\theta_{t}\right)\tilde{F}\left(\theta_{t}\right)\right)x\left(t\right), \end{array}$$

(95b) $$\begin{array}{cc} \; & \tilde{F}\left(i\right)=-{\left(B^{⊤}\left(i\right)E_{i}\left(\tilde{X}\right)B\left(i\right)\right)}^{-1}B^{⊤}\left(i\right)E_{i}\left(\tilde{X}\right)A\left(i\right), \end{array}$$

*is ESMS.*

Applying Theorem 5.13 from [3] in the DTARE case (94), we obtain the following.

**Corollary** **2.** *Under assumptions (**H3**) and (**H6**), the following are equivalent:*

*(i)*  *The DTARE (94) has a stabilizing solution $\tilde{X}$ satisfying the sign conditions $B^{⊤}\left(i\right)E_{i}\left(\tilde{X}\right)B\left(i\right)>0$, i∈Θ;*
*(ii)*  *The nominal system (83) is stochastic stabilizable and there exists $Y=\left(Y\left(1\right),\ldots ,Y\left(N\right)\right)\in S_{n}^{N}$ satisfying the following LMIs:*

(96) $$\left(\begin{array}{cc} A^{⊤}\left(i\right)E_{i}\left(Y\right)A\left(i\right)+\sum_{k=1}^{r}\alpha_{k}^{2}\left(i\right)C_{k}^{⊤}\left(i\right)C_{k}\left(i\right)-Y\left(i\right) & A^{⊤}\left(i\right)E_{i}\left(Y\right)B\left(i\right)\\ ★ & B^{⊤}\left(i\right)E_{i}\left(Y\right)B\left(i\right) \end{array}\right)>0,\;i\in \Theta .$$

The next result provides a comparison between the solutions of the LMIs in (91a) associated with some stabilizing feedback gains $\left(F(1),\cdots ,F(N)\right)$ and the stabilizing solution of the DTARE (94).

**Lemma** **3.** *(a) If X=(X(1),…,X(N)) is a solution of the LMI (91a) corresponding to a set of stabilizing gain matrices (F(1),…,F(N)) and to a vector of scaling parameters $\alpha_{k}\left(i\right)>0$, then we have $X\left(i\right)\geq \tilde{X}\left(i\right)$, i∈Θ, where $\tilde{X}=\left(\tilde{X}\left(1\right),\ldots ,\tilde{X}\left(N\right)\right)$ is the unique stabilizing solution of the DTARE (94) corresponding to the same set of parameters $\alpha_{k}\left(i\right)$, $\left(k,i\right)\in \underline{r}\times \Theta$.*

*(b)*  *If X=(X(1),…,X(N)) is a solution of (88a) satisfying (92), then $X\left(i\right)\geq \tilde{X}\left(i\right)$, i∈Θ.*

**Proof.**  (Hint) The DTARE (94) satisfied by $\tilde{X}$ is rewritten in the form

$$\begin{array}{cc} \tilde{X}\left(i\right) & ={\left(A\left(i\right)+B\left(i\right)F\left(i\right)\right)}^{⊤}E_{i}\left(\tilde{X}\right)\left(A\left(i\right)+B\left(i\right)F\left(i\right)\right)+\sum_{k=1}^{r}\alpha_{k}^{2}\left(i\right)C_{k}^{⊤}\left(i\right)C_{k}\left(i\right)\\ \; & -{\left(F\left(i\right)-\tilde{F}\left(i\right)\right)}^{⊤}\left(B^{⊤}\left(i\right)E_{i}\left(\tilde{X}\right)B\left(i\right)\right)\left(F\left(i\right)-\tilde{F}\left(i\right)\right), \end{array}$$

$\tilde{F}\left(i\right)$ being the same as in (95b). □

**Theorem** **5.** *Assume the following:*

*(a)*  *Assumptions (**H1**)–(**H3**) and (**H6**) are fulfilled;*
*(b)*  *The nominal system in (83) is stochastic stabilizable;*
*(c)*  *There exists a set of parameters $\alpha_{k}\left(i\right)>0$, $\left(k,i\right)\in \underline{r}\times \Theta$ for which the LMIs in (96) are solvable.*

*Let $\tilde{F}\left(i\right)$, i∈Θ be the gain matrices associated via (95b) with the stabilizing solution $\tilde{X}$ of the DTARE (94). Under these conditions, the control $u\left(t\right)=\tilde{F}\left(\theta_{t}\right)x\left(t\right)$ robustly stabilizes the nominal system in (83). The stability radius achieved by this control satisfies*

(97) $$\begin{array}{c} \rho_{D}\left[A+B\tilde{F},P|{\left(D_{k},C_{k}\right)}_{k\in \underline{r}}\right]\geq \sup\left\{\sigma >0|{\left(\frac{\sigma }{\alpha_{k}\left(i\right)}\right)}^{2}D_{k}^{⊤}\left(i\right)E_{i}\left(\tilde{X}\right)D_{k}\left(i\right)<I_{m_{k}},\left(k,i\right)\in \underline{r}\times \Theta \right\}. \end{array}$$

**Proof.**  The DTARE (94) satisfied by $\tilde{X}$ can be written as follows:

(98) $$\begin{array}{c} \tilde{X}\left(i\right)={\left(A\left(i\right)+B\left(i\right)\tilde{F}\left(i\right)\right)}^{⊤}E_{i}\left(\tilde{X}\right)\left(A\left(i\right)+B\left(i\right)\tilde{F}\left(i\right)\right)+\sum_{k=1}^{r}\alpha_{k}^{2}\left(i\right)C_{k}^{⊤}\left(i\right)C_{k}\left(i\right). \end{array}$$

Let σ>0 be arbitrary such that ${\left(\frac{\sigma }{\alpha_{k}\left(i\right)}\right)}^{2}D_{k}^{⊤}\left(i\right)E_{i}\left(\tilde{X}\right)D_{k}\left(i\right)<I_{m_{k}}$, $\forall \left(k,i\right)\in \underline{r}\times \Theta$; hence,

(99) $$∥{\left(\frac{\sigma }{\alpha_{k}\left(i\right)}\right)}^{2}D_{k}^{⊤}\left(i\right)E_{i}\left(\tilde{X}\right)D_{k}\left(i\right)∥<1,\;\forall \left(k,i\right)\in \underline{r}\times \Theta .$$

From (53), (89), (98), and (99) we deduce via Corollary 1(a) applied in the case of systems (85) and (86) that

(100) $$\rho_{D}\left[A+B\tilde{F},P|{\left(D_{k},C_{k}\right)}_{k\in \underline{r}}\right]>\sigma .$$

Thus, (97) is satisfied, since (100) is satisfied for an arbitrary σ>0 for which (99) holds. Thus, the proof is complete. □

**Remark** **8.** *(a) From Theorem 4, it can be concluded that the largest stability radius achieved by a control of type (84) with gain matrices computed via (90) based on a solution X of the matrix inequalities in (88a) corresponding to a vector of scaling parameters $\alpha_{k}\left(i\right)$, i∈Θ is provided by*

(101) $$\rho_{D}\left[A+B\hat{F},P|{\left(D_{k},C_{k}\right)}_{k\in \underline{r}}\right]\geq \sup\left\{\sigma >0|(88)\;\mathrm{are}\;\mathrm{fulfilled}\right\}.$$

*(b)*  *From Lemma 3,*
*we may infer that*
(102) $$\begin{array}{cc} \; & \left\{\sigma >0|{\left(\frac{\sigma }{\alpha_{k}\left(i\right)}\right)}^{2}D_{k}^{⊤}\left(i\right)E_{i}\left(X\right)D_{k}\left(i\right)<I_{m_{k}},\;\left(k,i\right)\in \underline{r}\times \Theta \right\}\subset \\ \; & \subset \left\{\sigma >0|{\left(\frac{\sigma }{\alpha_{k}\left(i\right)}\right)}^{2}D_{k}^{⊤}\left(i\right)E_{i}\left(\tilde{X}\right)D_{k}\left(i\right)<I_{m_{k}},\;\left(k,i\right)\in \underline{r}\times \Theta \right\} \end{array}$$
  *for all X=(X(1),…,X(N)) which solves the matrix inequalities in (88a) and for $\tilde{X}$ being the stabilizing solution of the DTARE (94).*
*(c)*  *Based on (97), (101), and (102), we may conclude that for a given set of scaling parameters $\alpha_{k}\left(i\right)>0$, $\left(k,i\right)\in \underline{r}\times \Theta$, the stability radius achieved by*
(103) $$\tilde{u}\left(t\right)=\tilde{F}\left(\theta_{t}\right)x\left(t\right),$$
  *where $\tilde{F}\left(i\right)$ is computed as in (95b), has a lower bound which is greater or equal to the lower bound of the stability radius achieved by any other control of type (84) having gain matrices computed as in (90) based on a solution of (88a). At the same time, it is worth mentioning that reliable numerical methods exist for numerically computing the gain matrices of controls of type (103).*

## 6. Numerical Example

**Figure 1:**
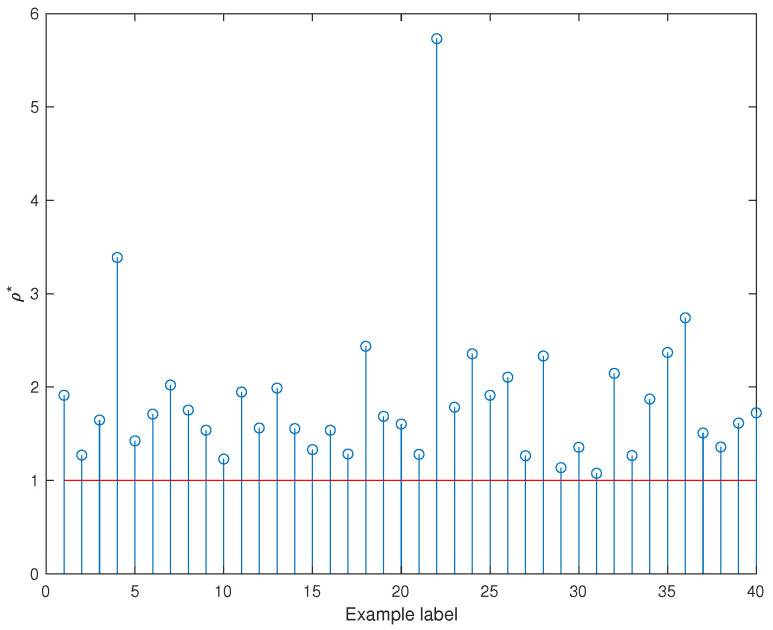
Plot of the quantity $\rho^{*}=\frac{\sigma_{\alpha }^{*}}{\sigma^{*}}$.

In this section, we consider the problem of robust state feedback stabilization in the time-invariant case using the results in Theorem 5. We show how the scaling technique allows for improved estimation of the lower bound of the stability radius when compared to a non-scaling technique. Let $\sigma^{★}$ and $\sigma_{\alpha }^{★}$ be the lower bounds corresponding to the non-scaling and scaling paradigms, respectively. We have randomly generated 40 numerical examples, then computed the parameter $\rho^{*}=\frac{\sigma_{\alpha }^{★}}{\sigma^{★}}$ for each example (Figure 1). It can be seen that the scaling technique paradigm improves the estimation of the stability radius of the considered class of stochastic uncertain systems.

## 7. Conclusions

In this paper, we have addressed the problem of robust stability analysis of a class of discrete-time time-varying Markovian jump linear systems subject to block-diagonal stochastic parameter perturbations. As a robustness measure, we have used the concept of stability radius, for which we obtain an estimation of its lower bound. We obtain a Riccati-based characterization allowing for efficient numerical computation of the stability radius. Our ongoing efforts are mainly devoted to extending the proposed framework to a more general class of systems, such as Markov regenerative switched linear systems, which generalize Markov and semi-Markov switching and allow for broader modeling flexibility

## Data Availability

Data are contained within the article.
